# Non-sedation versus sedation with a daily wake-up trial in critically ill patients receiving mechanical ventilation (NONSEDA Trial): study protocol for a randomised controlled trial

**DOI:** 10.1186/1745-6215-15-499

**Published:** 2014-12-20

**Authors:** Palle Toft, Hanne Tanghus Olsen, Helene Korvenius Jørgensen, Thomas Strøm, Helle Lykkeskov Nibro, Jacob Oxlund, Karl-Andre Wian, Lars Marius Ytrebø, Bjørn Anders Kroken, Michelle Chew

**Affiliations:** Department Anaesthesiology and Intensive Care, Odense University Hospital, Sdr. Boulevard 29, DK - 5000 Odense C, Denmark; Department Anaesthesiology and Intensive Care, Svendborg Hospital, Valdemarsgade 53, 5700 Svendborg, Denmark; Department Anaesthesiology and Intensive Care, Hospital Lillebaelt, Skovvangen 2-8, 6000 Kolding, Denmark; Department Anaesthesiology and Intensive Care, Aarhus University Hospital, Nørrebrogade 44, 8000 Aarhus, Denmark; Department Anaesthesiology and Intensive Care, Esbjerg Hospital, Frihedsvej 15, 6700 Esbjerg, Denmark; Department Anaesthesiology and Intensive Care, Vestfold Hospital, Halfdan Wilhelmsens alle 17, 3116 Tonsberg, Norway; Department Anaesthesiology and Intensive Care, University Hospital of North Norway, Sykehusvegen 38, 9019 Tromsoe, Norway; Department Anaesthesiology and Intensive Care, Hallands Hospital, Lasarettsvägen, 302 33 Halmstad, Sweden

**Keywords:** Critically ill patients, Non-sedation, Daily wake-up trial, Mechanical ventilation, Acute kidney injury, Delirium, Randomised controlled trial

## Abstract

**Background:**

Through many years, the standard care has been to use continuous sedation of critically ill patients during mechanical ventilation. However, preliminary randomised clinical trials indicate that it is beneficial to reduce the sedation level. No randomised trial has been conducted comparing sedation with no sedation, *a priori* powered to have all-cause mortality as primary outcome.

The objective is to assess the benefits and harms of non-sedation versus sedation with a daily wake-up trial in critically ill patients.

**Methods/Design:**

The non-sedation (NONSEDA) trial is an investigator-initiated, randomised, clinical, parallel-group, multinational trial designed to include 700 patients from at least six ICUs in Denmark, Norway and Sweden.

Inclusion criteria are mechanically ventilated patients with expected duration of mechanical ventilation >24 hours.

Exclusion criteria are non-intubated patients, patients with severe head trauma, coma at admission or status epilepticus, patients treated with therapeutic hypothermia, patients with PaO2/FiO2 < 9 where sedation might be necessary to ensure sufficient oxygenation or place the patient in prone position.

Experimental intervention is non-sedation supplemented with pain management during mechanical ventilation.

Control intervention is sedation with a daily wake-up trial.

The primary outcome will be all cause mortality at 90 days after randomization. Secondary outcomes will be: days until death throughout the total observation period; coma- and delirium-free days; highest RIFLE score; days until discharge from the intensive care unit (within 28 days); days until the participant is without mechanical ventilation (within 28 days); and proportion of patients with a major cardiovascular outcome. Explorative outcomes will be: all cause mortality at 28 days after randomisation; days until discharge from the intensive care unit; days until the participant is without mechanical ventilation; days until discharge from the hospital; organ failure.

Trial size: we will include 700 participants (2 × 350) in order to detect or reject 25% relative risk reduction in mortality with a type I error risk of 5% and a type II error risk of 20% (power at 80%).

**Discussion:**

The trial investigates potential benefits of non-sedation. This might have large impact on the future treatment of mechanically ventilated critically ill patients.

**Trial register:**

ClinicalTrials.gov NCT0196768, 09.01.2014.

**Electronic supplementary material:**

The online version of this article (doi:10.1186/1745-6215-15-499) contains supplementary material, which is available to authorized users.

## Background

### Patient population

**Figure 1:**
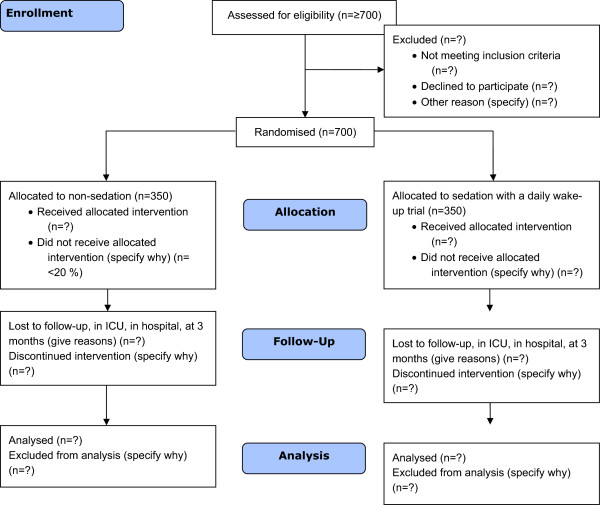
**CONSORT flowchart illustrating the randomization and flow of patients in the study.**

In Denmark approximately 30,000 patients (2 to 3% of all hospital patients) are admitted to intensive care units (ICUs) every year. Since treatment in ICUs is highly specialised and depends on technical equipment, like ventilators, it is costly and accounts for a large percentage of the total hospital expenditure. In 2011, 43% of the patients in Danish ICUs were medical patients, the rest surgical patients. The majority of the surgical patients were hospitalised acute. Mortality during the stay in ICU was 12.7% and 30-day mortality 21.2% [1]. An intensive care admission can have substantial consequences for patients and studies show that ICU survivors have a decreased quality of life and an increased mortality for years after discharge [2] (Figure 1).

### Current care and treatment

Since the dawn of ventilator therapy, it has been standard care to sedate the patients continuously. The first ventilators were rather primitive and highly uncomfortable for the patients, making the sedation practice necessary. Though ventilators are now advanced and allow for better patient-ventilator interaction, the routine of continuous sedation is still widespread. Continuous sedation has a reputation of being unavoidable for patients to tolerate the ICU environment, the necessary procedures and so forth. A study from USA in 1991 showed that almost all patients on ventilators were sedated, some by intermittent administration, while 62% received continuous intravenous sedative infusion [3]. A study from Scandinavia in 2004 showed that only 15% of the ICUs who answered the questionnaire applied daily awakening of the sedated patients [4]. A total of 53% of the ICUs used a sedation scale to monitor sedation depth. A study from Denmark in 1999 showed that sedatives were used extensively in Danish ICUs [5].

It has been shown that the level of melatonin in the blood, which is closely related to a proper diurnal sleep rhythm, is disturbed in sedated, mechanically ventilated patients [6]. This disturbed melatonin rhythm could play a role in the development of delirium, which is common in critically ill patients, with a prevalence of up to 80% [7].

During the last two decades, several randomised clinical trials have been conducted in an attempt to determine the adverse effects of continuous sedation during mechanical ventilation and to reduce the level of sedation in critically ill patients. The outcomes have primarily been ‘days on mechanical ventilation’ and ‘length of stay’ in ICU and in hospital. Brook *et al*. published a randomised clinical trial, which indicated that the use of protocol-controlled sedation could reduce the duration of mechanical ventilation, the length of stay in the ICU, and the number of tracheostomies performed [8].

In 1998, Kollef *et al*. performed a non-randomised cohort study, which showed that continuous intravenous infusion of sedatives was associated with significantly longer mechanical ventilation, increased length of stay in the ICU and in hospital, compared with patients receiving only bolus doses of sedatives [9]. In 2004, De Jonghe *et al*. showed that the use of a sedation algorithm, based on a regular assessments of the level of consciousness and an attempt to help the critically ill patients tolerate the intensive care environment, resulted in a significantly shorter duration of mechanical ventilation, using a historical control group [10].

In a trial from 2000, Kress *et al*. randomised 128 critically ill patients receiving mechanical ventilation to continuous intravenous sedation with or without a daily wake-up trial, in which the infusion of sedatives was discontinued [11]. When it had been ensured that the patient was co-operable and awake, the patient was put back to sleep using the continuous intravenous sedation. The trial showed that the group of patients who were awakened daily had a significantly shorter stay in the ICU and a significantly shorter duration of mechanical ventilation. Also, fewer diagnostic computed tomography (CT) scans of the cerebrum were performed in patients who were awakened daily [11]. Since Kress *et al*.’s trial it has been the golden standard to awaken patients on a daily basis if continuous intravenous sedation is used.

In 2005, Arroliga *et al*. carried out a prospective, multicentre, cohort study with 5,183 adult patients who were mechanically ventilated [12]. In this non-randomised study, it was observed that the use of sedatives for critically ill, mechanically ventilated patients was associated with longer duration of mechanical ventilation, a longer weaning period, and a longer stay in the ICU. It was concluded that randomised clinical trials specifically designed to evaluate the effect of sedation compared with no sedation should be performed on critically ill, mechanically ventilated patients to shed light on this important issue.

Most critically ill, mechanically ventilated patients in ICUs throughout the world are still routinely sedated. In the department of Anaesthesiology and Intensive Care at Odense University Hospital, we have had a non-sedation policy during the last decade. We use appropriate analgesics as intravenous bolus doses, but we do not sedate the patients. We undertook a randomised clinical trial to assess if this policy was in fact to the benefit of the patients [13]. One hundred and forty patients on mechanical ventilation were included and randomised to either the current golden standard of continuous sedation with a daily wake-up trial or to our standard therapy with no sedation. We were able to show that the non-sedated group had a significantly shorter time on mechanical ventilation (four days), a significantly shorter stay in the ICU (nine days), and a significantly shorter stay in hospital (24 days). The mortality was 22% in the non-sedated group and 38% in the sedated group (*P* = 0.06). A *post hoc* analysis demonstrated that the rate of acute renal failure was significantly lower in the non-sedated group [14]. A neuropsychologist examined a smaller cohort of the surviving patients. This psychological study demonstrated that the rate of post-traumatic stress was not significantly higher among patients who had been awake compared to those who were treated with the standard strategy of sedation [15].

To identify other randomised clinical trials assessing the effects of sedation versus non-sedation in mechanically ventilated, critically ill patients, we undertook a systematic literature search. We searched the following databases from inception to October 2013: Medline, Embase, Cinahl and the Cochrane Library, Central. Only one randomised clinical trial of sedation versus non-sedation was identified, namely the one we performed in Odense University Hospital [13].

The results from our previous trial showed that non-sedation in critically ill, mechanically ventilated patients might have substantial beneficial effects [13]. Our objective is now twofold: (1) to examine whether it is possible to implement non-sedation in other ICUs, and (2) to assess the clinical effects of non-sedation more thoroughly, randomising a sufficient number of patients to reach statistical power, to demonstrate or reject a significant reduction of mortality.

## Methods

### Trial conduct

We will obtain informed consent from the patients who are sufficiently awake; otherwise the informed consent will be obtained from the closest relative and the patient’s general practitioner, alternatively the Medical Health Office.

The trial is approved by the Data Protection Agency (#2008-58-0035, approval for the Region of Southern Denmark) and the regional ethics committee (Region Southern Denmark).

### Trial objectives and hypotheses

#### Objectives

The objective will be to assess the benefits and harms of the current golden standard of treatment, where mechanically ventilated ICU patients will be continuously sedated and awakened daily, compared with a strategy with non-sedation.

#### Hypotheses

The primary hypothesis is that non-sedation compared to sedation and a daily wake-up trial will:

- Reduce mortality.

The secondary hypotheses are that non-sedation compared to sedation and a daily wake-up trial will:

- Reduce the incidence of a composite outcome of death, acute myocardial infarction (AMI), stroke, pulmonary embolism and other thromboembolic events.
- Reduce the number of organ failures.
- Increase the days alive without mechanical ventilation.
- Increase the days alive outside the ICU.
- Increase the days alive outside the hospital.

### Trial design

The non-sedation (NONSEDA) trial is an investigator-initiated, randomised, clinical, parallel-group, multinational, superiority trial designed to include 700 patients from at least six ICUs in Denmark, Norway and Sweden (Additional files 1 and 2).

### Randomisation

Patients will be randomised to one of the two groups within 24 hours after intubation. If the patient arrives intubated from another ICU, the patient will be randomised within the first 24 hours after arrival. The participating doctors and study nurses will screen their ICU on a daily basis. The randomisation will be carried out centrally by the Copenhagen Trial Unit (CTU) according to a computer-generated allocation sequence with a variable block size, kept concealed from investigators at the clinical sites.

The allocation sequence will be stratified by centre, age (up to 65 years or older) and shock at admission (systolic blood pressure <70 mmHg or above).

The randomization system will be internet-based with 24 hours access a day, seven days a week. The participant allocation will be carried out by an investigator who logs in to the CTU’s online randomisation system using a personal ID and PIN code. Then the investigator will type in all relevant information about the participant, and the participant will be subsequently allocated to either the ‘non-sedation group’ or the ‘sedation group’.

### Blinding

Due to the nature of the trial interventions, it will not be possible to blind the investigators at clinical trial sites, the participants, and the participants’ relatives. All other parties in the trial will be blinded. Data regarding outcomes will be collected from national registers. The statistical analyses will be conducted blinded with the two intervention groups coded as, for example, A and B. Two conclusions will be drawn by the blinded steering committee; one assuming A is the experimental group and B in the control group, and one assuming the opposite. After that the code will be broken.

### Selection of participants

Patients will be included in the NONSEDA trial if they comply with the following inclusion and exclusion criteria:

Inclusion criteria

- Endotracheally intubated within the last 24 hours
- Expected time on ventilator >24 hours as estimated by the attending physician
- Age ≥18 years
- Informed consent

Exclusion criteria

- Severe head trauma where therapeutic coma is indicated
- Therapeutic hypothermia where therapeutic coma is indicated
- Status epilepticus where therapeutic coma is indicated
- Patient has participated in the study before
- Patient is transferred from another ICU with length of stay >48 hours
- Patient is comatose at admission
- Patient is brain dead
- The ratio of partial pressure arterial oxygen and fraction of inspired oxygen (PaO_2_/FiO_2_) ≤9, if sedation is necessary for oxygenation or prone position

### Discontinuation and withdrawal

#### Discontinuation and withdrawal at the choice of the participant or relative

A participant or a patient’s relative who no longer agrees to participate in the trial, can withdraw the informed consent at any time without need of further explanation. This will not have any consequences for the participant’s further treatment. In order to conduct intention-to-treat analyses with as little missing data as possible, it is in the interest of the trial to collect as much data from each participant as possible. Therefore, if possible, the investigator may ask the participant and/or relatives which aspects of the trial he/she wishes to withdraw from:

- receiving the trial intervention;
- participation in the remaining follow-up assessments (questionnaires and physical data);
- collection of data from registers;
- use of already collected data in the data analyses.

In Scandinavia, we have centrally registered the outcome of all patients. The only loss to follow-up will be patients who wish to withdraw their informed consent. Thus patients who, despite randomisation to non-sedation, do not receive the experimental intervention, is recorded as intention to treat. Their stay in the ICU will still be carefully recorded so that it subsequently will be possible to characterize the patients who poorly tolerate being awake. Specifically, it is recorded if failure of non-sedation is related to agitated delirium, oxygenation problems, or an unstable circulation. There will be no cross-over between the groups. If a patient in the non-sedated group needs sedation a daily wake-up trial will be performed. During this wake-up trial, it is evaluated if the patient again can tolerate non-sedation.

We expect that 15 to 20% of the patients in the non-sedated group will have difficulties tolerating the non-sedation strategy, mostly because of agitated delirium (18% discontinued the non-sedation in the previous study conducted at Odense University Hospital [13]).

### Selection of trial sites and personnel

#### Trial sites and setting

Odense University Hospital. Mixed ICU, medical and surgical patients, 26 beds

Lillebaelt Hospital, Kolding. Mixed ICU, medical and surgical patients, 11 beds.

Svendborg Hospital. Mixed ICU, medical and surgical patients, 7 beds.

Esbjerg Hospital. Mixed ICU, medical and surgical patients, 12 beds.

Aarhus University Hospital, Norrebrogade. Mixed ICU, medical and surgical patients, 14 beds.

Vestfold Hospital, Tønsberg. Mixed ICU, medical and surgical patients, 6 beds.

Tromsø University Hospital. Mixed ICU, medical and surgical patients, 10 beds.

Hallands Hospital, Halmstad. Mixed ICU, medical and surgical patients, 8 beds (Additional file 3).

#### Trial personnel

The trial personnel will be doctors and nurses in the selected ICUs. The personnel will be trained in non-sedation and daily wake-up trials, both in theory and by supervised practice.

### Trial interventions

#### Experimental intervention - non-sedation

The experimental group will not receive sedatives. Patients are thoroughly and repeatedly informed by the staff of where they are, what has happened, and what type of treatment they are going to receive.

If patients arrive at the ICU sedated and are randomised to the non-sedation group, the intravenous infusion of sedatives will be discontinued and the patient will be awakened.

Participants will be awake and have a natural sleep rhythm. In case these patients develop an agitated delirium, it is necessary to have a nurse or other caregiver at the bedside in order to calm the patient. Patients with delirium will be treated with haloperidol according to the U.S. guidelines, 2002 and the Danish national guidelines [16, 17].

If, despite these measures, it is necessary to sedate an agitated patient more than twice, or where sedation might be necessary to ensure sufficient oxygenation or to place the patient in a prone position, the patient will be sedated. Every day during the wake-up trial it is evaluated whether the patient is able to continue the non-sedation. There will be no cross-over between the groups.

In the previous trial from Odense, less than 18% of the included participants experienced failure of non-sedation (defined as sedation more than twice) [13].

### Control intervention - sedation with daily awakening attempt

The control group will be sedated with continuous infusion of sedatives to Ramsay score 3 to 4. The first 48 hours the patients will be sedated with propofol, after 48 hours midazolam will be used. This is in accordance with Danish and international practice and guidelines [16, 17]. During the daytime, the patient will be awakened as the intravenous infusion of sedatives will be discontinued. The patient will be considered to be awake when he/she can perform at least three of the following four tasks:

- Open the eyes to verbal commands.
- Follow the examiner's instructions with the eyes.
- Squeeze hands on request.
- Stick out the tongue on request.

After a successful wake-up, the infusion of sedative will be resumed, starting on half of the pre-wake-up dose. If the patient becomes uncomfortable or agitated during the awakening, sedation will be resumed, again starting with half the dosage. The infusion of sedatives will then be adjusted to Ramsey score 3 to 4.

Time zero is when the randomization is done.

### Co-interventions

Both intervention groups will receive analgesic treatment as usual, with opiates and paracetamol, in order to keep patients comfortable. In case the patient arrives at the department with an epidural catheter, the analgesia will continue via the epidural catheter as usual. The Visual Analogue Pain score (VAS) will be used to monitor the need for supplemental analgesics and morphine will be given if VAS is ≥3 at rest and ≥5 during activity.

All patients will be assessed for delirium on a daily basis, using the confusion assessment method in the ICU (CAM-ICU) score. If patients become agitated or delirious, they will be treated with haloperidol according to national [16] and international guidelines [17].

We have defined three co-interventions that will be registered and presented for each intervention group:

- Use of vasoactive agents
- Use of antibiotics
- Total amount of intravenous fluids (including blood products)
- Total amount of morphine and haloperidol

### Weaning from the ventilator

During the daily ward rounds it is estimated whether the patient meets the criteria to start weaning from the ventilator. As early as possible, the ventilator will be switched to pressure support ventilation. The pressure support will be reduced as much as possible guided daily by pCO_2_, tidal volume, and respiratory frequency.

If there are no signs of respiratory acidosis, a daily weaning trial will be performed. The pressure support will be reduced with 2 cm of water per hour. In the sedation group, the weaning trial will take place simultaneously with the wake-up trial. The patients will be awakened if the patient tolerates a weaning trial lasting 30 to 120 minutes without any signs of fatigue (signs of fatigue are shown as a respiratory rate >35, decreasing tidal volume, respiratory rate divided by tidal volume >105/minute, rising end tidal CO2 (ETCO2), saturation <90, paradoxical respiration, heart rate >140, systolic blood pressure >180 or <90).

Sedation will be discontinued when positive end-expiratory pressure (PEEP) = 5 and FiO_2_ = 40%.

The patient will be extubated and discharged from the ICU according to the departments’ clinical criteria.

Guidelines for extubation:

- Pressure Support (PS) <2
- PEEP <5
- FiO_2_ < 0.4
- Respiration frequency <35
- No CO_2_ retention
- Heart rate <140
- Adequate secretion quantity/acceptable cough force

### Waking the patient

If sedation duration is less than five days, the intravenous sedation will be discontinued immediately. If the intravenous sedation has lasted more than five days, it must be reduced gradually to avoid withdrawal symptoms. Eventually, sedatives with a long half-life can be exchanged with those with a short half-life in the hours before the patient is awakened. Possibly symptoms of withdrawal will be treated with clonidine.

### Compliance

Non-compliance to the protocol will be considered in the control group if drugs other than propofol and midazolam are used for sedation, if no wake-up trial is performed and if there is a major deviation from the Ramsey score 3 to 4.

Non-compliance to the protocol will be considered in the non-sedation group if the patients are sedated due to reasons other than agitated delirium, problems with the oxygenation or the need for the prone position.

The sponsor of the trial and the assigned postdoc will inspect the participating sites to ensure that the protocol is followed.

### Outcomes

The primary outcome will be:

- All-cause mortality at 90 days after randomisation.

Secondary outcomes will be:

- Days until death throughout the total observation period.
- Proportion of patients with a major cardiovascular outcome (acute myocardial infarction, cerebral infarction, cerebral haemorrhage, pulmonary embolus, deep vein thrombosis, other thromboembolic event) at 90 days after randomisation.
- Number of coma and delirium-free days (defined as Richmond agitation-sedation scale (RASS) ≥3 and no positive CAM-ICU scorings the particular day) within 28 days from randomisation
- Highest Risk, Injury, Failure, Loss of kidney function, and End-Stage kidney disease (RIFLE) score within 28 days from randomization

(RIFLE categories [18, 19]:

RIFLE-R: increase in serum creatinine × 1.5 from baseline OR urine output <0.5 mL/kg/hr × 6 h.

RIFLE-I: increase in serum creatinine × 2 from baseline OR urine output <0.5 mL/kg/hr × 12 h.

RIFLE-F: increase in serum creatinine × 3 from baseline OR urine output <0.3 mL/kg/hr × 24 h OR creatinine ≥350 μmol/L with acute rise ≥44 μmol/L in <24 h).

- Length of ICU stay, up to death or up to 28 days post randomization, whatever happens first
- Days until the participant is without mechanical ventilation (within 28 days from randomisation).

Exploratory outcomes are:

- All-cause mortality at 28 days after randomisation.
- Length of ICU stay, up to death or up to 90 days post randomization, whatever happens first.
- Days until the participant is without mechanical ventilation (within 90 days from randomisation).
- Length of hospital stay (within 90 days from randomisation).
- Organ failure when the patient is discharged from the ICU.
- Number of accidental extubations requiring re-intubation within 1 hour
- Number of accidental removals of central venous lines, requiring re-insertion within 4 hours

The monitoring of patients will be stopped 90 days after randomization.

### Safety

#### Definitions

Adverse event (AE): any undesirable medical event occurring to a participant during a clinical trial, which does not necessarily have a causal relationship with the intervention [20].

Serious adverse event (SAE): any adverse event that results in death, is life-threatening, requires hospitalisation or prolongation of existing hospitalisation, results in persistent or significant disability or incapacity, or is a congenital anomaly or birth defect [20].

Suspected unexpected serious adverse reaction (SUSAR): any suspected adverse reaction that is both serious and unexpected (unexpected means that the nature or severity of the event is not consistent with the information available to date) [20].

### Risks and safety issues in the NONSEDA trial

At the single-centre study at Odense University Hospital, the ICU diagnosed several patients with agitated delirium in the non-sedated group [13]. This difference might be caused by the fact that it is easy to discover agitated delirium in an awake patient. It is very difficult, if not impossible, to diagnose the same condition in a sedated patient.

In this multicentre trial, we will examine all patients for silent as well as agitated delirium by the use of CAM-ICU [16].

The sedated group will be awakened daily according to the Danish and international golden standard. It will thus be ensured, in both groups, that the participants will not be unduly heavily sedated with consequent prolonged admission.

### Recording and reporting of SAEs

As both the primary and the secondary outcomes all represent a range of possible SAEs, SAEs are estimated to occur very frequently in almost all patients. Therefore, only SAEs that are related to the study intervention will be recorded. These events will be accidental extubations requiring re-intubation within one hour, and accidental discontinuations of central venous accesses, which require reinsertion within four hours.

Investigators will report SAEs according to national standard operational procedures. Participating doctors and study nurses will screen for AEs. Screening is mandatory. SAEs will be registered in the electronic case record form (eCRF) and reported to the Data Monitoring and Safety Committee (DSMC) and Ethics Committee. The attending physician decides if an AE is serious.

### Procedures, assessments and data collection

#### Ethics

The study was approved by the Danish Ethics committee 09012014 (ID:S-20130025) and by the Norwegian Ethics committee 07052014 (ID :2013/2347/REK sor-ost).

### Inclusion of patients

Patients can be admitted to the ICU either from the same hospital (emergency department or another ward) or transferred from an ICU in another hospital. If they are admitted from within the same hospital, they are either not intubated or have been intubated within a very short time, for example during pre-hospital care. Patients will be included in the study within 24 hours post intubation.

Patients transferred from an ICU in another hospital are very often intubated. If they are transferred from another ICU, they can be included in the trial if the stay in the other ICU was shorter than 48 hours. In the time leading up to inclusion and randomisation, it will vary whether patients are sedated or not, depending on the particular clinician on duty and traditions at the particular hospital.

### Obtaining informed consent

When patients are contacted the first time concerning participation in the study, they will be at the ICU. Verbal and written information will be given by one of the participating physicians or study nurses. Patients are informed about the rights to assistance and the possibility of reflection time.

Patients will be considered competent if they are awake and not delirious (negative CAM-ICU). The competent patients will give consent after a period of reflection time of up to several hours.

If patients are not awake and not competent because of their illness, surrogate consent will be obtained from a close relative and the patient’s private practitioner, alternatively the Medical Health Office.

The consent of a relative relies on the patient’s presumed attitude to participation in clinical trials. The connection between the relative and the patient will appear in the surrogate consent form. Like the patient, the relative is also given time to make the decision of up to several hours.

If a patient or the relatives for any reason no longer wish to participate in the trial, they will be asked for permission to use the already obtained data, to obtain data from electronic patient files for the rest of the trial period, and to invite the patient to the 90-day follow-up (the latter only applies to patients in Kolding).

### Data collection

Table 1 shows the types of data and the time the data will be collected. If not otherwise stated, data originate from medical records included in the Critical Information System (CIS) or other patient files or observational cards.

**Table 1:** **Data registration time points**

	Day 1	After 1 day	Daily routine	At extubation	Discharge from ICU	Discharge from hospital	90 days after randomization
**Informed content**	X						
**Inclusion criteria**	X						
**Patient characteristics**	X						
**APACHE II, SOFA, SAPS II**		x					
**Organ effects**			X				
**CT- + MR cerebrum**			X				
**Unintended discontinuation of equipment**			X				
**The use of sedatives**			X				
**The use of morphine**			X				
**The time on mechanical ventilation**				X			
**Ventilator-associated pneumonia**				X			
**Length of stay, ICU**					X		
**Coma-free, delirium-free days**					X		
**Death in ICU**					X		
**Length of stay, hospital**						X	
**Death in hospital**						X	
**Exclusion**			X				
**Acute myocardial infarction**							X
**Thromboembolic complications**							X
**Death at 90 days**							X
**Serious adverse events**			X				

ICU, intensive care unit; APACHE II, acute physiology and chronic health evaluation II; SOFA, sequential organ failure assessment; SAPS II, simplified acute physiology score II; CT, computed tomography; MR, magnetic resonance.

### Data management

An eCRF for NONSEDA trial in Open Clinica is developed in cooperation between the coordinating investigator and a data manager at CTU (Copenhagen Trial Unit). Access to the eCRF will be possible around the clock every day where data can be entered continuously for all the randomised patients.

The coordinating investigator will have access to monitor the data input from all the participating centres.

If the entry is partially or completely missing or seems flawed on one or more randomised patients, the coordinator will have the opportunity to contact the primary investigator on the site in order to correct or complement data inputs to optimize the quality of the data.

### Statistics

#### Sample size estimation

In the single-centre study from Odense University Hospital, the mortality during hospitalization in the intention-to-treat analysis was 29% in the non-sedated group and 39% in the sedated group, corresponding to a relative risk reduction of 25% in hospital mortality [13].

In other studies and meta-analyses, the 90-day mortality of the corresponding populations of mechanically ventilated patients seems to be approximately 40% [21, 22]. It is therefore estimated, with a chosen maximal risk of type I error of 5% and risk of type II error of 20% (power = 80%) that 350 patients shall be randomised to each of the intervention groups, 700 patients in total, to show or reject a relative mortality reduction of 25%, absolute risk reduction of 10% corresponding to numbers needed to treat of 10. This sample size calculation has been made using the power and sample size program PS [23].

### Interim analysis

Study monitoring and interim analyses will be made by an independent DMSC. The DMSC has access to the data and will conduct their evaluation on the basis of these, when interim analysis is conducted.

The DSMC consists of a statistician and two senior doctors with extensive experience in intensive care medicine.

An interim analysis from the DMSC will be carried out when approximately 350 patients have been included. The committee will recommend to stop the trial if one of the groups show excess mortality or increased frequency of SAEs, corresponding to a *P* value at the interim analysis at 0.001 [24, 25], equal to Haybittle-Peto’s rule of discontinuing and/or Lan-DeMets terminating.

### Statistical analysis plan

All patients are followed up for at least three months after discharge via the eCRF, Social Security Register (SSR) and the National Patient Register (NPR). Missing data will be handled in accordance with multiple imputation procedures if missing data are greater than 5% and Little’s test is statistically significant [26]. Loss to follow-up is estimated to be minimal but all data will be analysed by intention to treat.

Primary outcome measure

All-cause mortality at 90 days after randomisation

Primary analysis. Multivariate logistic regression analysis adjusting for stratification variables [27].

Secondary analysis. Unadjusted univariate logistic regression.

Tertiary analysis. Multivariate logistic regression analysis adjusting for stratification variables and other design variables (simplified acute physiology score II (SAPS II) score, sequential organ failure assessment (SOFA) score, +/− shock at randomization, +/− chronic kidney disease, +/− chronic obstructive pulmonary disease (COPD), +/− daily benzodiazepine treatment prior to randomisation).

The secondary outcome measures:

1. Survival data will be analysed according to the following three analyses:
  Primary analysis: Cox-regression analysis adjusted for stratification variables given that proportional hazards are fulfilled, evaluated and judged by inspection of Log(−Log) and that these cumulative hazard curves are parallel for the intervention groups.
  Secondary analysis: unadjusted Cox-regression analysis, given that proportional hazard are fulfilled, evaluated and judged by inspection of Log(−Log) and that these cumulative hazard curves are parallel for the intervention groups, and illustrated with Kaplan-Meier curves.
  Tertiary analysis: Cox-regression analysis adjusted for the above-mentioned stratification variables and other design variables given that proportional hazard are fulfilled, evaluated by inspection of Log(−Log) and cumulative hazard curves by intervention group.
2. Dichotomous outcomes will be analysed using logistic regression (see the analysis plan for the primary outcome).
3. Continuous outcomes will be compared between the two intervention groups using the univariate general linear model. If the model assumptions cannot be fulfilled with reasonable approximation, a non-parametric test will be used (Mann-Whitney).
4. Count data will be analysed using van Elteren test (a stratified version of the nonparametric Wilcoxon rank-sum test) adjusting for site. Bootstrapping will be used to obtain a non-parametric estimate of the confidence interval of the intervention effect.

*P* values of less than 0.05 will be considered statistically significant, as the risk of statistical multiplicity in the assessment of the secondary endpoints will be evaluated [28]. Statistical analysis of the data will be done using SPSS 19 (IBM Corp, Armonk, NY, USA) and STATA 13 (StatCorp, College Station, TX, USA).

A detailed plan of analysis will be prepared before the mid-term interim analysis.

## Discussion

The trial investigates the potential benefit of non-sedation on clinically relevant endpoints. If a beneficial effect is shown, this would have a large impact on future treatment of mechanically ventilated critically ill patients.

## Trial status

Inclusion has started: 9 January 2014. Inclusion of patients is scheduled for completion 1 January 2017. Articles and PhD theses to be written and completed between 1 January 2017 and 1 June 2018. At present (July 2014) 55 patients have been included in the study.

## Electronic supplementary material

Additional file 1: **World Health Organization Trial registration.** Data set. (DOCX 19 KB)

Additional file 2: **Protocol revision chronology.** (DOCX 18 KB)

Additional file 3: **Organisational structure and responsibilities.** (DOCX 19 KB)

## Abbreviations

AMI: acute myocardial infarction
APACHE: acute physiology and chronic health evaluation
AE: adverse events
CIS: Critical Information System
CT: computed tomography
CTU: Copenhagen Trial Unit
COPD: chronic obstructive pulmonary disease
CAM-ICU: the confusion assessment method in the ICU
DMSC: Data Monitoring and Safety Committee
eCRF: electronic case record form
ETCO2: end-tidal carbon dioxide
ICU: intensive care unit
FiO_2_: fraction of inspired oxygen
MR: magnetic resonance
NPR: National Patient Registrar
NONSEDA: non-sedation
PaO_2_: partial pressure arterial oxygen
PEEP: positive end-expiratory pressure
PS: pressure support
RASS: Richmond agitation-sedation scale
RIFLE: Risk, Injury, Failure, Loss of kidney function, and End-Stage kidney disease (criteria for acute renal dysfunction)
SAE: serious adverse events
SAPS: simplified acute physiology score
SCR: Social Security Registrar
SOFA: sequential organ failure assessment
SOPs: standard operating procedures
SUSAR: suspected unexpected serious adverse reactions
VAS: visual analogue pain score.

## References

[1] **Danish intensive care database, DID** Available at: [https://www.sundhed.dk/content/cms/12/4712_did-årsrapport-2013-2014_18dec2014_endelig.pdf]. Accessed September 10, 2013

[2] Granja C, Amaro A, Dias C, Costa-Pereira A (2012). Outcome of ICU survivors: a comprehensive review. The role of patient-reported outcome studies. Acta Anaesthesiol Scand.

[3] Hansen-Flaschen J, Brazinsky S, Basile C, Lanken P (1991). Use of sedating drugs and neuromuscular blocking agents in patients requiring mechanical ventilation for respiratory failure. JAMA.

[4] Guldbrand P, Berggren L, Brattebö G, Mälstam J, Rönholm E, Winsö O (2004). Survey of routines for sedation of patients on controlled ventilation in Nordic intensive care units. Acta Anaesthesiol Scand.

[5] Christensen BV, Thunedborg LP (1999). Use of sedatives, analgesics and neuromuscular blocking agents in Danish ICUs 1996/97. A national survey. Intensive Care Med.

[6] Olofsson K, Alling C, Lundberg D, Malmros C (2004). Abolished circadian rhythm of melatonin secretion in sedated and artificially ventilated intensive care patients. Acta Anaesthesiol Scand.

[7] Ely EW, Shintani A, Truman B, Speroff T, Gordon SM, Harrell FE, Inouye SK, Bernard GR, Dittus RS (2004). Delirium as a predictor of mortality in mechanically ventilated patients in the intensive care unit. JAMA.

[8] Brook AD, Ahrens TS, Schaiff R, Prentice D, Sherman G, Shannon W, Kollef MH (1999). Effect of a nursing-implemented sedation protocol on the duration of mechanical ventilation. Crit Care Med.

[9] Kollef MH, Levy NT, Ahrens TS, Schaiff R, Prentice D, Sherman G (1998). Use of continuous iv sedation is associated with prolongation of mechanical ventilation. Chest.

[10] De Jonghe B, Bastuji-Garin S, Fangio P, Lacherade J-C, Jabot J, Appéré-De-Vecchi C, Rocha N, Outin H (2005). Sedation algorithm in critically ill patients without acute brain injury. Crit Care Med.

[11] Kress JP, Pohlman AS, O’Connor MF, Hall JB (2000). Daily interruption of sedative infusions in critically ill patients undergoing mechanical ventilation. NEJM.

[12] Arroliga A, Frutos-Viver F, Hall J, Esteban A, Apezteguí C, Soto L, Anzueto A (2005). Use of sedatives and neuromuscular blockers in a cohort of patients receiving mechanical ventilation. Chest.

[13] Strøm T, Martinussen T, Toft P (2010). A protocol of no sedation for critically ill patients receiving mechanical ventilation: a randomised trial. Lancet.

[14] Strøm T, Johansen RR, Prahl JO, Toft P (2011). Sedation and renal impairment in critically ill patients: a post hoc analysis of a randomized trial. Crit Care.

[15] Strøm T, Stylsvig M, Toft P (2011). Long-term psychological effects of a no-sedation protocol in critically ill patients. Crit Care.

[16] **Danish Association of Anaesthesiology and Intensive Care, DASAIM. Guideline, sedation strategies** 2011.http://www.dasaim.dk/wp-content/uploads/2014/02/Sedationsstrategi_samlet.pdf

[17] Jacobi J, Fraser GL, Coursin DB, Riker RR, Fontaine D, Wittbrodt ET, Chalfin DB, Masica MF, Bjerke HS, Coplin WM, Crippen DW, Fuchs BD, Kelleher RM, Marik PE, Nasraway SA, Murray MJ, Peruzzi WT, Lumb PD (2002). Clinical practice guidelines for the sustained use of sedatives and analgesics in the critically ill adult. Crit Care Med.

[18] Kellum JA, Bellomo R, Ronco C (2007). The concept of acute kidney injury and the RIFLE criteria. Contrib Nephrol.

[19] Myburgh JA, Finfer S, Bellomo R, Billot L, Cass A, Gattas D, Glass P, Lipman J, Liu B, McArthur C, McGuinness S, Rajbhandari D, Taylor CB, Webb SA (2012). Hydroxyethyl starch or saline for fluid resuscitation in intensive care. N Engl J Med.

[20] International Conference on Harmonisation (2001). ICH harmonised tripartite guideline: Guideline for Good Clinical Practice. J Postgrad Med.

[21] Afshari A, Wetterslev J, Brok J, Møller A (2007). Antithrombin III in critically ill patients: systematic review with meta-analysis and trial sequential analysis. BMJ.

[22] Perner A, Haase N, Guttormsen AB, Tennhunen J, Klemenzson G, Aaneman A, Madsen KR, Møller MH, Elkjær JM, Poulsen LM, Bendtsen A, Winding R, Steensen M, Berezowicz P, Søe-Jensen P, Bestle M, Strand K, Wiis J, White JO, Thornberg KJ, Quist L, Nielsen J, Andersen LH, Holst LB, Thormar K, Kjældgaard AL, Fabritius ML, Mondrup F, Pott FC, Møller TP (2012). Hydroxyethyl starch 130/0.42 versus Ringer’s acetate in severe sepsis. N Engl J Med.

[23] Dupont WD, Plummer WD (1990). Power and sample size calculations. A review and computer program. Control Clin Trials.

[24] Haybittle JL (1971). Repeated assessment of results in clinical trials of cancer treatment. Br J Radiol.

[25] Lan K, DeMets D (1983). Discrete sequential boundaries for clinical trials. Biometrika.

[26] Schafer JL (1999). Multiple imputation: a primer. Stat Methods Med Res.

[27] Kahan BC, Morris TP (2012). Reporting and analysis of trials using stratified randomisation in leading medical journals: review and reanalysis. BMJ.

[28] Dmitrienko A, Soulakova JN, Millen BA (2011). Three methods for constructing parallel gatekeeping procedures in clinical trials. J Biopharm Stat.

