# Supplementary material for: Non-sedation versus sedation with a daily wake-up trial in critically ill patients receiving mechanical ventilation (NONSEDA Trial): study protocol for a randomised controlled trial
Source: Trials. 2014 Dec 20;15:499. doi: 10.1186/1745-6215-15-499 (PMC4307177; doi:10.1186/1745-6215-15-499)
Supplement: Supplementary file 1 — Additional file 1: World Health Organization Trial registration. Data set. (DOCX 19 KB) [file 13063_2014_2371_MOESM1_ESM.docx]

# Additional file 1 – World Health Organization Trial Registration. Data Set

| **Data category** | **Information** |
| --- | --- |
| Primary registry and trial identifying number | Clinical Trials Gov. Registration: NCT0 1967 680 |
| Date of registration in primary registry | 09.01.2014 |
| Secondary identifying numbers | DNVK: S2013 0025 |
| Source(s) of monetary or material support | The Danish Council for Strategic Research, Danielsen’s Foundation, Scandinavian Society of Anaesthesiology and Intensive Care Medicine |
| Primary sponsor | Palle Toft, Professor, MDSc, Dept. of Anaesthesiology and Intensive Care Medicine, Odense University Hospital, Odense, Denmark |
| Secondary sponsors | N-A |
| Contact for public queries | Palle Toft, Professor, MDSc, Dept. of Anaesthesiology and Intensive Care Medicine, Odense University Hospital, Odense, Denmark  Tel.: +45 6541 3947  Email: [palle.toft@rsyd.dk](mailto:palle.toft@rsyd.dk) |
| Contact for scientific queries | Palle Toft, Professor, MDSc, Dept. of Anaesthesiology and Intensive Care Medicine, Odense University Hospital, Odense, Denmark  Tel.: +45 6541 3947  Email: [palle.toft@rsyd.dk](mailto:palle.toft@rsyd.dk) |
| Public title | The NONSEDA-trial |
| Scientific title | Non-sedation versus sedation with a daily wake-up trial in critically ill patients receiving mechanical ventilation – the NONSEDA trial |
| Countries of recruitment | Denmark, Norway, Sweden |
| Health condition(s) or problem(s) studied | Non-sedation to critically ill mechanical ventilated patients, mortality, delirium, acute kidney injury, lenght of mechanical ventilation, ICU length of stay, hospital length of stay, major cardiovascular outcome, organ failure, number of accidental extubation, accidental removals of central venous lines |
| Intervention(s) | Treatment arm: Non sedation supplemented with pain management during mechanical ventilation.  Control arm: Sedation with a daily wake-up trial supplemented with pain management during mechanical ventilation. |
| Key inclusion and exclusion criteria | Inclusion criteria: Mechanical ventilated patients with expected duration of mechanical ventilation > 24 hours.  Exclusion criteria: Non intubated patients, patients with severe head traumer, coma at admission or status epilepticus, patients treated with therapeutic hypothermia, patients with PAO2/FiO2 < 9 where sedation might be necessary, to ensure sufficient oxygenation or to place the patient in prone position. |
| Study type | Interventional  Allocation: prospective randomized  Intervention model: double arm  Masking: not blinded (obvious if the patient is sedated or awake)  Primary purpose: treatment |
| Date of first enrolment | January 2014 |
| Target sample size | 700 |
| Recruitment status | Recruiting |
| Primary outcome | Mortality |
| Key secondary outcomes | Days until death throughout the total observation period, coma and delirium free days, highest Rifle-score score, days until discharge from the intensive care unit (within 28 days from randomisation), days until the participant is without mechanical ventilation (within 28 days), proportion of patients with a major cardiovascular outcome (excluding death)  Explorative outcomes will be: All cause mortality at 28 days after randomization, days until discharge from the intensive care unit (within 90 days), days until the participants is without mechanical ventilation (within 90 days), days until discharge from the hospital (within 90 days), organ failure, number of accidental extubations requiring re-intubation within 1 hour, number of accidental removals of central venous lines, requiring re-insertion within 4 hours |
|  |  |
