# Supplementary material for: Non-sedation versus sedation with a daily wake-up trial in critically ill patients receiving mechanical ventilation (NONSEDA Trial): study protocol for a randomised controlled trial
Source: Trials. 2014 Dec 20;15:499. doi: 10.1186/1745-6215-15-499 (PMC4307177; doi:10.1186/1745-6215-15-499)
Supplement: Supplementary file 2 — Additional file 2: Protocol revision chronology. (DOCX 18 KB) [file 13063_2014_2371_MOESM2_ESM.docx]

# Additional file 2 - Protocol revision chronology

Issue Date 09.01.2014

Protocol Amendment Number: 02

Authors: Palle Toft MD, DMSc

Hanne Tanghus, MD

Helene Korvenius Jørgensen, MD

Thomas Strøm, MD, Ph.D.

Helle Nibro, MD, Ph.D.

Jakob Oxlund, MD

Karl-Andre Wian, MD

Michelle Chew, MD, Ph.D.

Lars Marius Ytrebø, MD, Ph.D., Tromsø

Kroken Bjørn Anders MD

| Date | Protocol Amendment |
| --- | --- |
| Original Version, 21.02.2013  Version 1 was the first version to be submitted to the Danish Scientific Ethical Committee | Amendment No. 1  Primary reason for amendment: At the request of the Danish Scientific Ethical Committee, information sheet for patients and relatives were changed. |
| Version 2, 24.06.2013 | Amendment No. 2  Primary reason for amendment:The economic support for the trial was highlighted as requested by the Danish Scientific Ethical Committee. |
| 09.01.2014 | Amendment No. 3  Primary reason for amendment: The treatment in the control group was changed. Patients, who, despite randomization to non-sedation, do not receive the experimental intervention, will be recorded as intention to treat.  Their stay in the ICU will still be carefully recorded. Every day during the wake-up trial it will be evaluated if the patient is able to continue with non-sedation. |
