# Supplementary material for: Non-sedation versus sedation with a daily wake-up trial in critically ill patients receiving mechanical ventilation (NONSEDA Trial): study protocol for a randomised controlled trial
Source: Trials. 2014 Dec 20;15:499. doi: 10.1186/1745-6215-15-499 (PMC4307177; doi:10.1186/1745-6215-15-499)
Supplement: Supplementary file 3 — Additional file 3: Organisational structure and responsibilities. (DOCX 19 KB) [file 13063_2014_2371_MOESM3_ESM.docx]

# Additional file 3 – Organisational structure and responsibilities

| Title | Name | Function |
| --- | --- | --- |
| Principal Investigator | Palle Toft, MD, DMSc | Design and conduct of NONSEDA trial .  Protocol preparation and revisions  Preparation of Case Report Forms  Managing Clinical Trials Office  Publication of study reports  Member of both committees  Organization steering committee meetings |
| Lead Investigators | Hanne Tanghus, MD, Svendborg Hospital  Helene Korvenius Jørgensen, MD, Lillebælt Hospital  Thomas Strøm, MD, Ph.D., Odense University Hospital  Helle Nibro, MD, Ph.D., Aarhus University Hospital  Jakob Oxlund, MD, Esbjerg Central Hospital  Karl-Andre Wian, MD Vestfold Hospital  Michelle Chew, MD, Ph.D., Hallands Hospital  Lars Marius Ytrebø MD,ph.d. and  Anders Bjørn Kroken, MD, Tromsø University Hospital | Maintain trial master file  Resolve contractual issues at sites  Responsible for identification, recruitment, data collection and completion of CRF’s along with follow up of study patients and adherence to study protocol |
| Steering committee | Palle Toft, MD,DMSc  Hanne Tanghus, MD  Helene Korvenius Jørgensen, MD  Thomas Strøm, MD, Ph.D. | Agreement of final protocol  Reviewing progress of study  Approving changes to the protocol  Budget administration  Advice for lead investigator |
| Trial Management Committee | Palle Toft, MD,DMSc  Hanne Tanghus, MD  Helene Korvenius Jørgensen, MD  Thomas Strøm, MD, Ph.D. | Decide when site visit will occur  Data verification  Randomization  Provide annual risk report to the Danish Scientific Ethical Committee  Serious adverse events reporting |
| Data Manager | Ema Erkocevic | Maintenance of trial IT system and data verification |
